# Comparison of postoperative pain and stress using a multimodal approach in cats: open vs. laparoscopic-assisted ovariohysterectomy

**DOI:** 10.3389/fvets.2024.1519773

**Published:** 2024-12-19

**Authors:** Changwoo Jeong, Kangwoo Yi, Yong Yu, Suyoung Heo

**Affiliations:** Department of Surgery, College of Veterinary Medicine, Jeonbuk National University, Iksan-si, Republic of Korea

**Keywords:** cats, feline laparoscopic surgery, minimally invasive surgery, laparoscopic-assisted ovariohysterectomy, postoperative pain evaluation

## Abstract

**Introduction:**

Laparoscopic surgery is increasingly utilized for its minimally invasive nature, leading to reduced postoperative pain and faster recovery. This study aimed to compare postoperative pain, surgical complications, and recovery between laparoscopic-assisted ovariohysterectomy (LAOHE) and open ovariohysterectomy (OHE) in cats.

**Methods:**

A total of 40 healthy female cats were randomly assigned to undergo either LAOHE (*n* = 20) or OHE (*n* = 20). Pain scores were assessed using the Glasgow Composite Pain Scale at 1, 4, 8, 12, and 24 h postoperatively. Blood samples were collected to measure cortisol levels as a stress biomarker. Complications were recorded intraoperatively and postoperatively.

**Results:**

Cats in the LAOHE group exhibited significantly lower pain scores compared to the OHE group at 1, 4, and 8 h postoperatively (*P* < 0.05). Cortisol levels were also significantly lower in the LAOHE group (*P* < 0.05). There were no significant differences in surgical time or postoperative complications between the two groups.

**Discussion:**

The findings suggest that LAOHE results in reduced postoperative pain and stress in cats compared to OHE, without increasing surgical time or complications. LAOHE may be a preferable technique for elective ovariohysterectomy in cats.

## 1 Introduction

Elective ovariohysterectomy (OHE) is a routine surgical procedure commonly performed in general veterinary practice, with its benefits and potential complications extensively documented (1). Elective OHE can be performed using either a ventral median celiotomy or a bilateral flank laparotomy approach (2). Laparoscopic surgery is a well-established technique in feline veterinary practice, especially for elective procedures (3–5). Laparoscopic procedures, including laparoscopic-assisted OHE (LAOHE), have recently gained popularity, particularly in reproductive and gastrointestinal surgeries, and have demonstrated feasibility in canine patients (6–8). Laparoscopic-assisted techniques retain the minimally invasive advantages of laparoscopy while allowing complex tasks to be completed more efficiently through extracorporeal methods (8). Factors such as the extent of soft tissue trauma, duration of surgery, and phrenic nerve irritation likely influence pain intensity (9–14). In dogs, LAOHE is associated with improved peri-operative visualization, reduced stress and pain, lower morbidity, and faster recovery (11). Although these benefits are anticipated in cats, their smaller size can pose additional challenges, making laparoscopic surgery more complex and time-intensive (15, 16). Furthermore, laparoscopic equipment incurs significant costs and requires specialized technical support for routine use. Compared with dogs, fewer studies have examined pain associated with laparoscopic surgery in cats, likely because of the difficulty of assessing pain in this species (17).

To date, no well-controlled study has directly compared postoperative pain in cats undergoing laparoscopic-assisted vs. traditional open ovariohysterectomy. This study therefore aimed to compare the duration of surgery, surgical complications, stress, and pain between LAOHE and open OHE in a controlled prospective randomized clinical trial. We hypothesized that, as observed in dogs, LAOHE would result in less postoperative pain, stress, and inflammation than open OHE, while showing comparable duration of surgery and complication rates.

## 2 Materials and methods

### 2.1 Animal selection

This study was approved by the Institutional Animal Care and Use Committee of Jeonbuk National University. All procedures were conducted in accordance with the animal use and ethics guidelines at Jeonbuk National University.

Thirty healthy, sexually intact female cats (26 Korean Shorthairs, two Scottish Folds, and two Russian Blues) awaiting adoption through local animal shelters were randomly assigned to either the OHE or LAOHE groups. Prior to the study, consent was obtained from the shelter director for all cats involved. Health status was confirmed through history, physical examination, and hematologic testing including preoperative complete blood count, venous blood gas analysis, and serum biochemistry [feline serum amyloid A (SAA), cortisol, and glucose]. Seven days before surgery, the cats were admitted and handled by a designated surgeon to reduce stress, particularly for those sensitive to postoperative hospitalization. This approach aimed to achieve stable and accurate cortisol and glucose levels by minimizing stress-related fluctuations. Cats displaying aggressive behavior, those experiencing uncontrolled pain postoperatively requiring rescue analgesia, and those with significant deterioration in health status during the study period were planned for exclusion. Food and water were provided until the night before surgery.

### 2.2 Anesthesia

Ten minutes after premedication with intravenous butorphanol (0.2 mg/kg) and midazolam (0.2 mg/kg), anesthesia was induced using intravenous propofol until the desired anesthetic effect was achieved. Anesthesia was maintained with sevoflurane delivered via endotracheal intubation with 100% oxygen. The systolic, diastolic, and mean arterial pressures were continuously monitored using oscillometry. Perioperative monitoring included electrocardiography (ECG), systolic blood pressure (SBP), heart rate (HR), respiratory rate (RR), end-tidal (ET) CO_2_, ET sevoflurane, pulse oximetry, and body temperature (BT), all of which were recorded throughout anesthesia.

Lactated Ringer's solution was administered intravenously at 5 mL/kg/h throughout surgery, with intravenous cefazolin (22 mg/kg) given immediately before the procedure. Mechanical ventilation was initiated if ETCO_2_ exceeded 55 mmHg during anesthesia.

### 2.3 Surgical procedures

All open and laparoscopic procedures were performed by a single surgeon, assisted by 1–2 experienced assistants and an anesthesiologist (Y.Y.). The ventral abdomen was clipped caudally from the xiphoid to the inguinal region. Each cat was then positioned on the surgical table or patient positioning device in dorsal recumbency with the hindlimbs extended. The skin was prepped with a chlorhexidine-ethanol solution (Chlorhexidine Gluconate 0.5% in 70% Ethanol Solution, Green Cross Corp., Yongin, South Korea).

#### 2.3.1 Open ovariohysterectomy group (OHE group)

A ventral median celiotomy was performed through a 2–3 cm incision in the skin and linea alba. The spay hook was inserted into the cranial end of the incision and press it against the left ventrolateral peritoneal surface. Rotated the hook toward the midline and straighten the handle so that it is perpendicular to the ventral abdominal wall. Slowly lift the hook and extract it from the abdomen. A laparotomy sponge was carefully placed within the peritoneal cavity to secure the left uterine horn and expose the proper ligament. Subsequently, mosquito forceps were applied to the proper ligament to manipulate the ovary. After elevating the ovary, the suspensory ligament was carefully transected. The broad ligament was punctured, and two Kelly forceps were positioned at the mesovarian pedicle. A 3-0 polyglactin 910 ligature was placed beneath each forcep on the pedicle, and the pedicle was transected between the ligatures using a bipolar device (mar-Clamp Cut IQ, KLS Martin Group, Tuttlingen, Germany). The uterine horn was followed to the contralateral side, and the suspensory ligament of the second ovary was transected. The second ovarian pedicle was ligated in the same manner as previously described. The uterine body was gently exteriorized to expose the bifurcation, and a ligature was placed around the uterine body just above the cervix and below the bifurcation. A clamp was applied between the bifurcation and ligatures, and the uterine body was transected between the clamp and ligatures using a bipolar device. The abdominal wall and subcutaneous tissue were closed with a simple continuous pattern using PDS II sutures (3-0, Ethicon, Johnson & Johnson, USA). The skin was closed with a simple interrupted suture pattern using nylon (3-0, Blue Nylon, AILEE co., Korea).

#### 2.3.2 Laparoscopic-assisted ovariohysterectomy group

**Figure 1:**
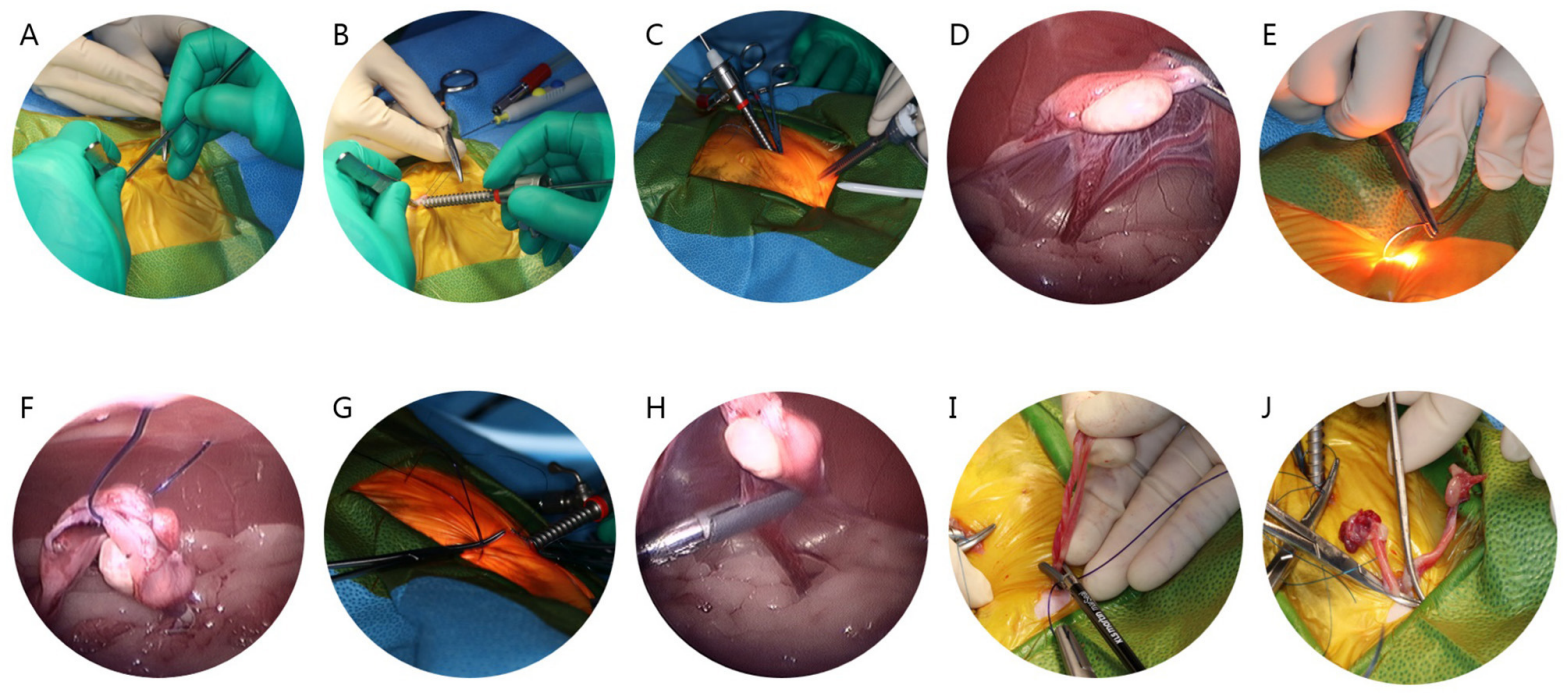
Stepwise surgical procedure for laparoscopic-assisted ovariohysterectomy in cats. **(A)** Use of a blunt K-wire instead of the style. **(B)** Cannula insertion. **(C)** Placement of the instrument portal under transabdominal illumination. **(D)** Retraction and elevation of the proper ligament of the right ovary toward the abdominal wall. **(E)** Confirmation of percutaneous suspension suture placement by transabdominal illumination. **(F)** Laparoscopic image during the percutaneous suspension suture. **(G)** External securing of the suspension suture using a hemostat. **(H)** Sealing of the ovarian pedicle with a vessel sealing device (VSD). **(I)** Exteriorization of both uterine horns through the instrument port. **(J)** Ligation and sealing caudal to the uterine bifurcation with a VSD.

While the cat was in dorsal recumbency, pneumoperitoneum was achieved using a modified Hasson technique with CO_2_ (at a pressure of 5–7 mm Hg). A 5–6 mm midline skin incision was made to establish the camera portal, located 1–1.5 cm caudal to the umbilicus. A blunt K-wire served as a substitute for the stylet, and a 6.5 cm long, 3.3 mm threaded cannula (Ternamian EndoTip, Karl Storz Endoscopy, Tuttlingen, Germany) was inserted (Figure 1A). Once the cannula was in place, the abdomen was insufflated with CO_2_ to a maximum pressure of 7 mmHg (Figure 1B). After the introducing a 3 mm, 30° Hopkins II laparoscope (Karl Storz Veterinary Endoscopy), the instrument portal was established under transabdominal illumination (Figure 1C). This second portal was established 4–5 cm caudal to the umbilicus using a disposable 6 mm threaded trocar-cannula assembly. Following the placement of the portal, the cat was tilted 25–30° laterally using a positioner to facilitate the identification of the right ovary. The proper ligament of the right ovary was retracted and lifted toward the abdominal wall using a 5 mm endoscopic bipolar vessel sealing device (VSD; marSeal 5 Plus, KLS Martin Group, Tuttlingen, Germany; Figure 1D). A suspension suture using PDS II sutures (0, Ethicon, Johnson & Johnson, USA) was performed percutaneously after confirming its location through transabdominal illumination (Figures 1E, F). The suspension suture was externally secured close to the abdominal wall using a hemostat, and the ovarian pedicle was sealed with a VSD (Figures 1G, H). The same procedure was repeated on the opposite side. After sealing both ovarian pedicles, the suspension suture was held with the VSD, and both uterine horns were exteriorized sequentially through the instrument port (Figure 1I). Finally, ligation was performed 0.5–1 cm caudal to the uterine bifurcation and sealed with the VSD (Figure 1J). After removing all ports, the abdominal wall was closed using a cruciate suture pattern, the subcutaneous tissue was sutured with a simple interrupted pattern using PDS II sutures (3-0, Ethicon, Johnson & Johnson, USA). The skin was closed with a simple interrupted pattern using nylon (3-0, Blue Nylon, AILEE co., Korea).

### 2.4 Monitored variables and postoperative pain assessment

Surgical and postoperative complications were documented for all procedures. Surgical time and anesthesia duration were recorded for each cat, with surgical time defined as the period from the first incision to the final skin or portal suture, and anesthesia duration as the period from induction to endotracheal extubation. The cats were assessed for HR, RR, BT, SBP, and pain scores at 1, 2, 4, 8, 12, and 24 h after extubation, all evaluated by a designated observer. In addition, meloxicam (0.2 mg/kg) was administered subcutaneously immediately before extubation, with additional doses (0.05 mg/kg) provided every 24 h for up to 3 days. Additionally, butorphanol (0.2 mg/kg) was administered 2 h after extubation. Blood samples were collected at 1, 2, 4, 8, 12, 24, 48, and 72 h postoperatively. Glucose and serum cortisol levels were measured for up to 24 h to evaluate postoperative stress, whereas SAA levels were measured for up to 72 h to assess the extent of postoperative tissue damage (18, 19). Serum cortisol levels were analyzed externally through IDEXX Laboratories, glucose was measured internally with a Dri-Chem NX700 (FUJIFILM Corporation, Tokyo, Japan), and SAA levels were assessed using the FUJI-IMMUNO AU10V (FUJIFILM Corporation, Tokyo, Japan). Postoperative pain intensity was initially scored on a 0–20 scale using the Glasgow Feline Composite Measure Pain Scale (CMPS-Feline) (20–22) (Appendix). Rescue analgesia was administered as needed based on a pain score exceeding 6, with intravenous morphine (0.1 mg/kg) given accordingly.

### 2.5 Statistical analysis

All statistical analyses were performed using GraphPad Prism (version 9.0.0; GraphPad Software, San Diego, CA, USA). The mean and standard deviation (SD) values were calculated for all measured variables. The normality of the data was evaluated using the Shapiro-Wilk test, and all variables met the assumption of normality (*p* > 0.05). Bartlett's test indicated that the assumption of homogeneity of variances was met for all variables (*p* > 0.05).

Age, weight, and surgical time were compared between treatment groups using an unpaired *t*-test. A two-way repeated-measures analysis of variance (ANOVA) was applied to assess the effects of treatment group and time on pain scores, followed by Tukey's multiple comparisons test for *post-hoc* analysis.

Physiological parameters, including glucose, cortisol, SAA, BT, SBP, HR, and RR, were analyzed using a one-way repeated-measures ANOVA with Dunnett's multiple comparisons test for *post-hoc* comparisons.

Statistical significance was set at *P* < 0.05. To account for the increased risk of Type I Error due to multiple comparisons, the *p*-values were adjusted using the Benjamini-Hochberg method.

## 3 Results

### 3.1 Study population

Thirty healthy female cats without gross reproductive abnormalities were included in this study, and equally assigned to the OHE and LAOHE groups. No cases met the exclusion criteria, and all enrolled subjects completed the study. The mean age was 3.36 ± 1.07 years in the OHE group and 4.05 ± 1.2 years in the LAOHE group (Table 1). The mean body weight was 3.75 ± 1.1 kg for the OHE group and 4.23 ± 1.26 kg for the LAOHE group (Table 1). No significant differences were observed between groups for age (*P* = 0.108) or body weight (*P* = 0.276).

**Table 1:** Measured quantitative data of the two studied groups.

**Variable**	**LAOHE**	**OHE**	***P*-value**
Age (years)	4.05 ± 1.2	3.36 ± 1.07	0.108
Weight (kg)	4.23 ± 1.26	3.75 ± 1.1	0.276
Surgical time (min)	42.6 ± 5	36.7 ± 11.9	0.088

LAOHE, laparoscopic-assisted ovariohysterectomy; OHE, open ovariohysterectomy.

### 3.2 Anesthesia and surgical procedures

No anesthetic or surgical complications occurred, and no conversions to the open approach were needed in the LAOHE group. The mean surgical time was similar between groups: 36.7 ± 11.9 min in the OHE group and 42.6 ± 5.0 min in the LAOHE group (P = 0.088; Table 1).

### 3.3 Recorded variables

No significant changes were observed over time in any measured vital parameters in either group. The results are summarized in Table 2.

**Table 2:** Time-dependent analysis of physiological parameters in cats undergoing laparoscopic-assisted ovariohysterectomy (LAOHE) and open ovariohysterectomy (OHE).

**Physiological parameter**	**LAOHE (*P*-value; time wise)**	**OHE (*P*-value; time wise)**
Body temperature (°C)	0.292	0.078
Systolic blood pressure (mmHg)	0.208	0.489
Heart rate (bpm)	0.265	0.127
Respiratory rate (bpm)	0.442	0.091

### 3.4 Hematological parameters

The blood tests were performed at 1, 2, 4, 8, 12, and 24 h after extubation to assess surgical stress and tissue damage intensity (Table 3). Blood glucose levels significantly increased from preoperative values at 1, 2, and 4 h in the OHE group and at 1 h in the LAOHE group.

**Table 3:** Cortisol, blood glucose, and serum amyloid A (SAA) concentrations (mean ± standard deviation) in cats undergoing laparoscopic-assisted ovariohysterectomy (LAOHE) and open ovariohysterectomy (OHE), measured at baseline and at 1, 2, 4, 8, 12, and 24 h after extubation, with SAA additionally measured at 48 and 72 h.

**Variable**	**Group**	**Preoperative**	**1 h**	**2 h**	**4 h**	**8 h**	**12 h**	**24 h**	**48 h**	**72 h**
Cortisol (μg/dL)	OHE	2 ± 1.1	3.6 ± 1.4^*^	4.1 ± 1.8^*^	4.7 ± 3.3	4.8 ± 3.9	3.3 ± 2.5	3.5 ± 3.1	-	-
	LAOHE	1.8 ± 1	2.9 ± 1.2	2.1 ± 1.8	1.9 ± 1.2	1.4 ± 0.9	1.8 ± 1.4	2.1 ± 1.2	-	-
Glucose (mg/dL)	OHE	92 ± 6.5	139.8 ± 19.8^*^	128.2 ± 14.7^*^	116 ± 24.4^*^	114 ± 25.6	105 ± 23.7	108 ± 24.1	-	-
	LAOHE	94.3 ± 6.9	121.9 ± 18.2^*^	106.7 ± 15.8	106.2 ± 14.2	109.1 ± 13.8	94.5 ± 11.3	96.3 ± 7.4	-	-
SAA (μg/mL)	OHE	5.2 ± 2.8	4.7 ± 2.4	10.3 ± 7.5	13.9 ± 9.6	27.5 ± 14.9^*^	33.8 ± 14^*^	31.5 ± 25.7^*^	12.4 ± 9.7	7.2 ± 4.1
	LAOHE	4.5 ± 2.7	6.4 ± 3.3	6.8 ± 5.2	9 ± 8.1	14.1 ± 8.9^*^	19.9 ± 9.7^*^	22.1 ± 13.4^*^	11.1 ± 7.4	9.3 ± 6.9

^*^Statistically significant difference (p < 0.05).

Cortisol levels showed a significant rise in the OHE group up to 2 h postoperatively compared with baseline values, with no significant change observed in the LAOHE group. SAA levels significantly increased in both groups from 8 to 24 h postoperatively.

### 3.5 Postoperative pain

One hour after extubation, all cats were fully conscious, with no significant difference in levels of consciousness levels observed between the two groups. Eight hours after extubation, no significant difference in appetite was observed between the groups. No cats required rescue analgesia.

**Figure 2:**
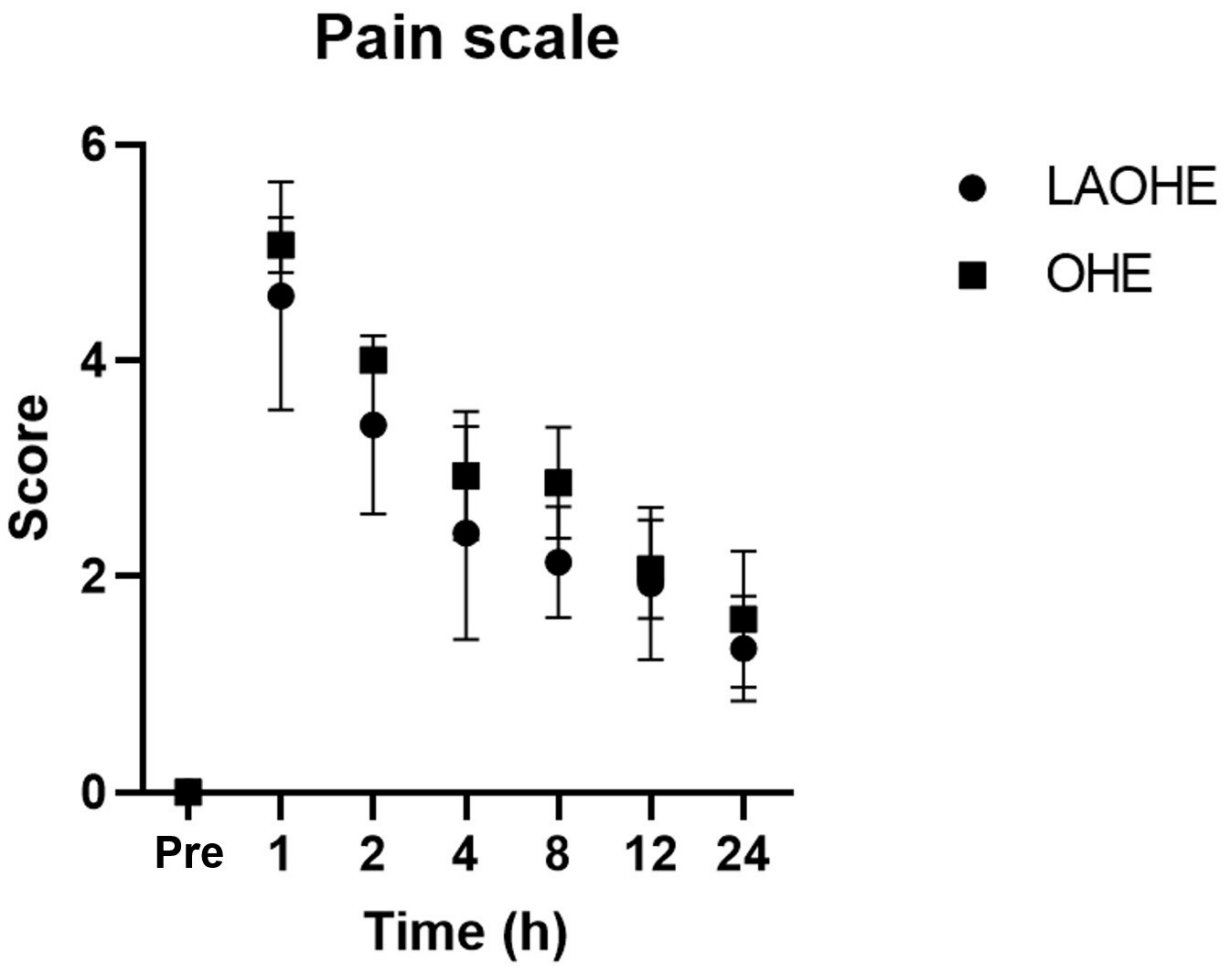
Pain scores (mean ± standard deviation) recorded at various intervals after extubation in cats undergoing either open ovariohysterectomy (circles) or laparoscopic-assisted ovariohysterectomy (squares).

The results of the two-way repeated-measures ANOVA indicated a significant difference in pain scale scores between the two groups (*P* = 0.029; Figure 2). *Post hoc* analysis revealed significant differences at the following postoperative time points: 1 h (*P* = 0.041), 2 h (*P* = 0.032), and 4 h (*P* = 0.017).

## 4 Discussion

Our study demonstrated that both LAOHE and OHE are safe and effective methods for elective ovariohysterectomy in cats; however, LAOHE resulted in significantly lower postoperative stress and pain. The differences in cortisol levels and pain scores between the two groups indicate that LAOHE is less invasive and stressful for patients, consistent with the findings of previous canine studies (7). In contrast to earlier research, such as that by Alves et al. (9), which did not observe significant differences in pain scores between the laparoscopic and open techniques, our study consistently found lower pain scores in the LAOHE group.

Our study addressed the limitations of previous research by carefully controlling for confounding factors such as environmental conditions, circadian variations, and standardized analgesic protocols, all of which are known to influence cortisol levels as a primary marker of stress during surgery (23, 24). By ensuring that all surgeries were conducted at the same time of day and that cats were acclimated to handling conditions a week before surgery, we minimized stress-induced fluctuations in cortisol levels. A significant increase in postoperative cortisol levels was observed in the OHE group, indicating that this procedure induced greater stress. This finding supports our hypothesis that the minimally invasive nature of LAOHE results in lower postoperative stress, reinforcing the benefits of laparoscopic surgery in feline patients.

In previous studies, pain assessments often relied on unidimensional scales such as the Simple Descriptive Scale and the Visual Analog Scale. Those scales can be subject to interpretation and lack sensitivity in capturing the full complexity of pain (25, 26). Moreover, they also do not provide a clear clinical threshold for administering additional analgesia, limiting their applicability in managing postoperative pain. To address these shortcomings, we used the CMPS-Feline scale, a validated multidimensional scale that integrates both behavioral and physiological indicators of pain (20–22). This scale offers a more comprehensive evaluation and includes a defined cutoff point for the administration of rescue analgesia. For these reasons, we utilized the CMPS-Feline scale in this study to assess pain in cats, as it may serve as a valuable tool for pain evaluation; however, further studies are required to validate its applicability.

The technical aspects of the procedure contributed significantly to the improved outcomes observed in our study. Previous comparisons of the laparoscopically assisted ovariectomy technique, which required three incision sites, and OHE did not demonstrate significant differences in pain scores or stress markers such as cortisol (4). In contrast, our study employed a two-port technique for LAOHE, which is associated with reduced tissue damage owing to fewer entry points. This modification likely played a crucial role in the significant reduction of pain and stress observed in the LAOHE group. The reduced invasiveness of the two-port technique supports the hypothesis that minimizing the number of surgical ports can limit trauma and, consequently, enhance postoperative outcomes. This finding underscores the importance of refining surgical techniques to further reduce stress and pain, particularly in laparoscopic procedures.

SAA was selected as a biomarker to assess the intensity of tissue damage in view of its sensitivity as a marker of acute inflammation (27). Although we observed a trend toward higher SAA levels in the OHE group, the differences were not statistically significant. This lack of significance may be attributed to the relatively short duration of our postoperative observation period, as previous research indicates that continuous monitoring of SAA over an extended period may yield more accurate insights into tissue damage (27, 28). Despite the lack of statistical significance, the overall higher SAA levels in the OHE group suggest that the more invasive nature of open surgery may result in greater tissue damage. This finding aligns with those of studies in other species, where open procedures typically cause more trauma than minimally invasive techniques (29). However, further research is necessary to better understand the role of SAA in detecting tissue damage in feline patients. Future studies should consider longer observation periods and larger sample sizes to fully assess its clinical utility.

This study has several limitations. The relatively small sample size may have limited the ability to detect subtle differences between the groups, highlighting the need for future studies with larger cohorts. The results were reported without adjustments for multiple comparisons to prioritize their clinical relevance; however, this approach may increase the potential for Type I error and warrants careful interpretation. Furthermore, the focus was on short-term recovery outcomes within 72 h, and long-term complications or benefits were not assessed. Further research is recommended to validate these findings and explore long-term outcomes.

## 5 Conclusion

In conclusion, our study demonstrates that two-port LAOHE significantly reduces postoperative pain and stress in cats compared with traditional OHE, as assessed using a multimodal approach. Although no notable difference in surgical time was observed between the two procedures, LAOHE minimizes tissue trauma, resulting in improved postoperative outcomes. These findings support LAOHE as the preferred technique for elective surgery in feline patients. As laparoscopic technology and expertise become more widely accessible, LAOHE should be considered a superior alternative in routine practice. Future research should focus on long-term postoperative recovery and further refine multimodal assessments to optimize the benefits of this technique.

## Data Availability

The original contributions presented in the study are included in the article/Supplementary material, further inquiries can be directed to the corresponding author.
