# Corrigendum: Comparison of postoperative pain and stress using a multimodal approach in cats: open vs. laparoscopic-assisted ovariohysterectomy

**DOI:** 10.3389/fvets.2025.1558304

**Published:** 2025-02-10

**Authors:** Changwoo Jeong, Kangwoo Yi, Yong Yu, Suyoung Heo

**Affiliations:** Department of Surgery, College of Veterinary Medicine, Jeonbuk National University, Iksan-si, Republic of Korea

**Keywords:** cats, feline laparoscopic surgery, minimally invasive surgery, laparoscopic-assisted ovariohysterectomy, postoperative pain evaluation

In the published article, there was an error. While the study exclusively involves cats, the term dogs was mistakenly used in the Abstract. The body of the manuscript is accurate and unaffected by this issue, but the inconsistency in the abstract could lead to confusion among readers.

A correction has been made to the **Abstract**. The Abstract previously stated:

“**Introduction:** Laparoscopic surgery is increasingly utilized for its minimally invasive nature, leading to reduced postoperative pain and faster recovery. This study aimed to compare postoperative pain, surgical complications, and recovery between laparoscopic-assisted ovariohysterectomy (LAOHE) and open ovariohysterectomy (OHE) in dogs.

**Methods:** A total of 40 healthy female dogs were randomly assigned to undergo either LAOHE (n = 20) or OHE (n = 20). Pain scores were assessed using the Glasgow Composite Pain Scale at 1, 4, 8, 12, and 24 h postoperatively. Blood samples were collected to measure cortisol levels as a stress biomarker. Complications were recorded intraoperatively and postoperatively.

**Results:** Dogs in the LAOHE group exhibited significantly lower pain scores compared to the OHE group at 1, 4, and 8 h postoperatively (P < 0.05). Cortisol levels were also significantly lower in the LAOHE group (P < 0.05). There were no significant differences in surgical time or postoperative complications between the two groups.

**Discussion:** The findings suggest that LAOHE results in reduced postoperative pain and stress in dogs compared to OHE, without increasing surgical time or complications. LAOHE may be a preferable technique for elective ovariohysterectomy in dogs.”

The corrected **Abstract** appears below:

“**Introduction:** Laparoscopic surgery is increasingly utilized for its minimally invasive nature, leading to reduced postoperative pain and faster recovery. This study aimed to compare postoperative pain, surgical complications, and recovery between laparoscopic-assisted ovariohysterectomy (LAOHE) and open ovariohysterectomy (OHE) in cats.

**Methods:** A total of 40 healthy female cats were randomly assigned to undergo either LAOHE (*n* = 20) or OHE (*n* = 20). Pain scores were assessed using the Glasgow Composite Pain Scale at 1, 4, 8, 12, and 24 h postoperatively. Blood samples were collected to measure cortisol levels as a stress biomarker. Complications were recorded intraoperatively and postoperatively.

**Results:** Cats in the LAOHE group exhibited significantly lower pain scores compared to the OHE group at 1, 4, and 8 h postoperatively (*P* < 0.05). Cortisol levels were also significantly lower in the LAOHE group (*P* < 0.05). There were no significant differences in surgical time or postoperative complications between the two groups.

**Discussion:** The findings suggest that LAOHE results in reduced postoperative pain and stress in cats compared to OHE, without increasing surgical time or complications. LAOHE may be a preferable technique for elective ovariohysterectomy in cats.”

The authors apologize for this error and state that this does not change the scientific conclusions of the article in any way. The original article has been updated.

