# Supplementary material for: Comparison of postoperative pain and stress using a multimodal approach in cats: open vs. laparoscopic-assisted ovariohysterectomy
Source: Front Vet Sci. 2024 Dec 19;11:1519773. doi: 10.3389/fvets.2024.1519773 (PMC11693733; doi:10.3389/fvets.2024.1519773)
Supplement: Supplementary file 1 [file Data_Sheet_1.pdf]

Glasgow Feline Composite Measure Pain Scale: CMPS - Feline

Choose the most appropriate expression from each section and total the scores to calculate the pain score for the cat. If more than one expression applies choose the higher score

LOOK AT THE CAT IN ITS CAGE:

Is it?

Question 1

Silent / purring / meowing	0
Crying/growling / groaning	1

Question 2

Relaxed	0
Licking lips	1
Restless/cowering at back of cage	2
Tense/crouched	3
Rigid/hunched	4

Question 3

Ignoring any wound or painful area	0
Attention to wound	1

Question 4

a) Look at the following caricatures. Circle the drawing which best depicts the cat's ear position?

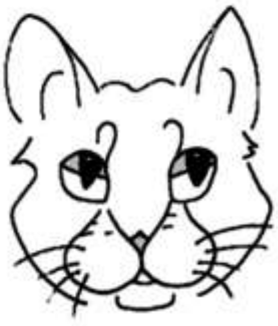

0

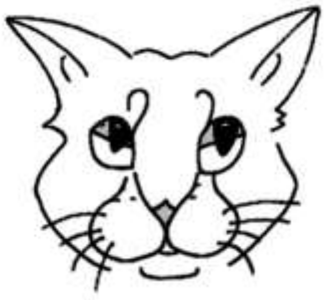

1

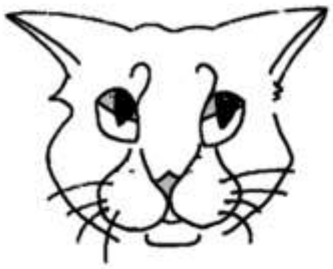

2

b) Look at the shape of the muzzle in the following caricatures. Circle the drawing which appears most like that of the cat?

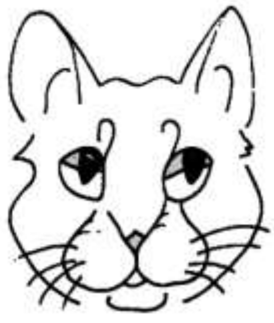

0

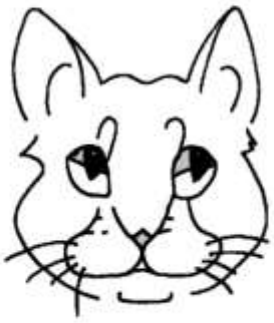

1

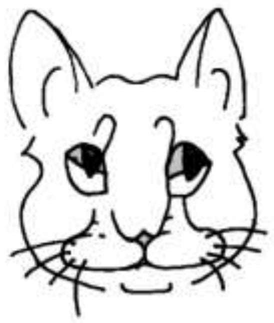

2

APPROACH THE CAGE, CALL THE CAT BY NAME & STROKE ALONG ITS BACK FROM HEAD TO TAIL

Question 5

Does it?

Respond to stroking	0

Is it?

Unresponsive	1
Aggressive	2

IF IT HAS A WOUND OR PAINFUL AREA, APPLY GENTLE PRESSURE 5 CM AROUND THE SITE. IN THE ABSENCE OF ANY PAINFUL AREA APPLY SIMILAR PRESSURE AROUND THE HIND LEG ABOVE THE KNEE

Question 6

Does it?

Do nothing	0
Swish tail/flatten ears	1
Cry/hiss	2
Growl	3
Bite/lash out	4

Question 7

General impression

Is the cat?

Happy and content	0
Disinterested/quiet	1
Anxious/fearful	2
Dull	3
Depressed/grumpy	4

Pain Score … /20

© Universities of Glasgow & Edinburgh Napier 2015. Licensed to NewMetrica Ltd. Permission granted to reproduce for personal and educational use only. To request any other permissions please contact jacky.reid@newmetrica.com.
